# What Am I Measuring? Disentangling Cognitive Status From Sensory Impairment

**DOI:** 10.1093/geroni/igaa057.2997

**Published:** 2020-12-16

**Authors:** Emma Nichols, Bonnielin Swenor, Jennifer Deal, Alison Abraham, Michelle Carlson, Pradeep Ramulu, Susan Resnick, Alden Gross

**Affiliations:** 1 Johns Hopkins Bloomberg School of Public Health, Baltimore, Maryland, United States; 2 Johns Hopkins University, Baltimore, Maryland, United States; 3 Johns Hopkins School of Medicine, Baltimore, Maryland, United States; 4 National Institute on Aging, Bethesda, Maryland, United States

## Abstract

Some studies have shown sensory impairment is associated with impaired cognitive test performance, however tests rely to varying degrees on hearing and vision. We hypothesized scores for cognitive tests whose administration depends on vision or hearing are biased among those with vision or hearing impairment, respectively, after controlling for underlying cognitive performance. We used item response theory methods to test for differential item functioning (DIF) by hearing and vision impairment in the Baltimore Longitudinal Study of Aging (BLSA) and the Atherosclerosis Risk in Communities study (ARIC). We identified DIF by hearing impairment for tests that do not rely on hearing and DIF by vision impairment for tests that do not rely on vision. We found no clinically meaningful differences between unadjusted and DIF-adjusted measures of cognition. Our finding that DIF by sensory domain was independent of administration modality suggests cognitive load may play a larger role than previously acknowledged.

